# Acacetin suppresses the electrocardiographic and arrhythmic manifestations of the J wave syndromes

**DOI:** 10.1371/journal.pone.0242747

**Published:** 2020-11-24

**Authors:** José M. Di Diego, Bence Patocskai, Hector Barajas-Martinez, Virág Borbáth, Michael J. Ackerman, Alexander Burashnikov, Jérôme Clatot, Gui-Rong Li, Victoria M. Robinson, Dan Hu, Charles Antzelevitch

**Affiliations:** 1 Lankenau Institute for Medical Research, Wynnewood, PA, United States of America; 2 Department of Cardiology, Angiology and Pulmonology, University Hospital, Heidelberg University, Heidelberg, Germany; 3 First Department of Medicine, Medical Faculty Mannheim, Heidelberg University, Mannheim, Germany; 4 Divisions of Heart Rhythm Services and Pediatric Cardiology, Departments of Cardiovascular Medicine, Pediatric and Adolescent Medicine, and Molecular Pharmacology & Experimental Therapeutics, Windland Smith Rice Genetic Heart Rhythm Clinic and Windland Smith Rice Sudden Death Genomics Laboratory, Mayo Clinic, Rochester, MN, United States of America; 5 Sidney Kimmel Medical College, Thomas Jefferson University, Philadelphia, PA, United States of America; 6 Xiamen Cardiovascular Hospital, Medical School of Xiamen University, Xiamen, China; 7 The University of Manchester, Manchester, United Kingdom; 8 Department of Cardiology and Cardiovascular Research Institute, Renmin Hospital of Wuhan University, Wuhan, China; 9 Lankenau Heart Institute, Wynnewood, PA, United States of America; University of Minnesota, UNITED STATES

## Abstract

**Background:**

J wave syndromes (JWS), including Brugada (BrS) and early repolarization syndromes (ERS), are associated with increased risk for life-threatening ventricular arrhythmias. Pharmacologic approaches to therapy are currently very limited. Here, we evaluate the effects of the natural flavone acacetin.

**Methods:**

The effects of acacetin on action potential (AP) morphology and transient outward current (I_to_) were first studied in isolated canine RV epicardial myocytes using whole-cell patch clamp techniques. Acacetin’s effects on transmembrane APs, unipolar electrograms and transmural ECGs were then studied in isolated coronary-perfused canine RV and LV wedge preparations as well as in whole-heart, Langendorff-perfused preparations from which we recorded a 12 lead ECG and unipolar electrograms. Using floating glass microelectrodes we also recorded transmembrane APs from the RVOT of the whole-heart model. The I_to_ agonist NS5806, sodium channel blocker ajmaline, calcium channel blocker verapamil or hypothermia (32°C) were used to pharmacologically mimic the genetic defects and conditions associated with JWS, thus eliciting prominent J waves and provoking VT/VF.

**Results:**

Acacetin (5–10 μM) reduced I_to_ density, AP notch and J wave area and totally suppressed the electrocardiographic and arrhythmic manifestation of both BrS and ERS, regardless of the experimental model used. In wedge and whole-heart models of JWS, increasing I_to_ with NS5806, decreasing I_Na_ or I_Ca_ (with ajmaline or verapamil) or hypothermia all resulted in accentuation of epicardial AP notch and ECG J waves, resulting in characteristic BrS and ERS phenotypes. Phase 2-reentrant extrasystoles originating from the RVOT triggered VT/VF. The J waves in leads V1 and V2 were never associated with a delay of RVOT activation and always coincided with the appearance of the AP notch recorded from RVOT epicardium. All repolarization defects giving rise to VT/VF in the BrS and ERS models were reversed by acacetin, resulting in total suppression of VT/VF.

**Conclusions:**

We present experimental models of BrS and ERS capable of recapitulating all of the ECG and arrhythmic manifestations of the JWS. Our findings provide definitive support for the repolarization but not the depolarization hypothesis proposed to underlie BrS and point to acacetin as a promising new pharmacologic treatment for JWS.

## Introduction

Acacetin is a natural flavone produced by several plants, including snow lotus, which is used in traditional Chinese medicine for multiple purposes, including treatment of rheumatoid arthritis, impotence, irregular menses, asthma, bronchitis, cough, and altitude sickness [1].

J wave syndromes (JWS) are characterized by distinctive J waves and/or ST segment elevation in certain ECG-leads and are associated with an increased risk for ventricular tachycardia (VT)/ventricular fibrillation (VF), leading to sudden cardiac death (SCD). The two principal forms of JWS are Brugada syndrome (BrS) and early repolarization syndrome (ERS). BrS is characterized by prominent J waves, often referred to as ST segment elevation, in the right precordial leads whereas ERS is characterized by J waves in the inferior and infero-lateral ECG leads. Thus, the substrate for BrS is in the right ventricle (RV), particularly in the right ventricular outflow tract (RVOT), whereas the region most involved in ERS is the inferior wall of the left ventricle (LV). However, in type 3 ERS, J waves appear globally, in the inferior, lateral, and anterior (right precordial) ECG leads, so that both ventricles are affected [2]. First line therapy for high-risk patients suffering from JWS is an implantable cardioverter defibrillator (ICD), but the approach is problematic in very young infants for whom a pharmacological alternative is desirable. Adjunct pharmacological therapy is also needed for individuals receiving frequent, appropriate, VF-terminating shocks. Finally, alternative pharmacological therapy is desirable for treatment of asymptomatic patients considered to be at high risk for a sentinel cardiac event [3]. For these reasons, there is a pressing need for specific pharmacologic approaches for primary or secondary prevention of arrhythmic events.

The *KCND3-*encoded Kv4.3 transient outward potassium current (I_to_) exerts a pivotal role in the pathophysiology of both syndromes [4,5], owing to its ability to accentuate the action potential (AP) notch and to suppress the AP dome. Li et al [1] reported that in atria, acacetin inhibits I_to_, the ultra-rapid delayed rectifier potassium current (I_Kur_), and the acetylcholine-activated potassium current (I_K-ACh_), without significantly influencing other major cardiac ion channel currents. These authors demonstrated its efficacy in preventing the induction of atrial fibrillation (AF) in an *in vivo* canine model. In the ventricle, acacetin selectively inhibits I_to_. The co-existence of AF in BrS patients is common (10–20%) and is positively correlated with the inducibility of ventricular arrhythmias [6]. Patients with a more advanced stage of BrS are more likely to have a history of atrial arrhythmias (27% vs 13% of BrS patients with vs. without an ICD indication) [7]. Therapeutic options targeting both BrS and AF would be of great benefit for these individuals.

Because I_Kur_ and I_K-ACh_ are negligible (or absent) in ventricular myocardium, our hypothesis is that acacetin could serve as a highly selective I_to_-blocker in the ventricles with the additional advantage of having no adverse effects, unlike quinidine, which is currently the pharmacologic treatment of choice for JWS. An ion-channel specific and cardio-selective I_to_ blocker has long been sought for the management of JWS [8]. The present study tests the hypothesis that acacetin, via its action to inhibit I_to_, can effectively suppress the electrocardiographic and arrhythmic manifestations of both BrS and ERS in experimental models designed to pharmacologically mimic the genetic defects and ion channel heterogeneities underlying these JWS. A secondary aim of the study was to create a whole-heart model of JWS with which to advance our understanding of the mechanisms underlying the electrocardiographic and arrhythmic manifestations of the JWS.

## Materials and methods

The Lankenau Institute for Medical Research Institutional Animal Care and Use Committee reviewed and approved this research.

We evaluated the effect of acacetin in: 1) HEK293 cells expressing human Kv4.3 and Nav1.5 using patch clamp techniques (37°C temp.), 2) canine ventricular epicardial myocytes 3) coronary-perfused canine right (RV) and left ventricular (LV) wedge models and 4) Langendorff-perfused canine whole-heart models of ERS and/or BrS in which the sodium channel blocker ajmaline or the calcium channel blocker verapamil, together with the transient outward current agonist NS5806 were used to provoke the arrhythmic phenotypes. BrS and ERS phenotypes were created by pharmacologically mimicking the genetic defects underlying these syndromes.

This investigation conforms to the Guide for Care and Use of Laboratory Animals. Twenty five purpose-bred mongrel dogs of either sex were acquired from Marshall BioResources (North Rose, NY), ranging from 9 to12 months of age (25–30 kg). Dogs were given ketamine (10 mg/kg, IM) and xylazine 2 (mg/kg, IM) for sedation prior to euthanasia. Once the dogs were sedated, they were anticoagulated with heparin (human pharmaceutical grade, 1,000 U/kg, IV) and euthanized with Euthasol solution (pentobarbital sodium and phenytoin sodium; 0.22 ml/kg, IV) via a paw vein. After the Euthasol was administered, we checked for the absence of a palpebral reflex, lack of withdrawal from a noxious stimulus applied to the distal forelimb, lack of breathing, and absence of auscultable heart rate before excision of the heart via a left thoracotomy. The hearts were then placed in cold cardioplegic solution (modified Tyrode’s or Krebs-Henseleit solutions containing 16 mM KCl).

Preparations consisting of RV and LV wedge preparations were isolated from the canine hearts. The coronary artery in each preparation was cannulated with Tyrode’s solution. With continuous coronary perfusion, all ventricular branches of the right coronary artery were immediately clamped and tied off with a silk thread. The total time from excision of the heart to cannulation and perfusion of the artery was <4 min. The preparations were placed in a temperature-controlled bath and perfused at a rate of 8–10 ml/min using Tyrode’s solution. The composition of the Tyrode’s solution was (in mM): NaCl 129, KCl 4, NaH_2_PO_4_ 0.9, NaHCO_3_ 20, CaCl_2_ 1.8, MgSO_4_ 0.5, and D-glucose 5.5, bubbled with 95% O_2_ and 5% CO_2_ (37±0.5 C, pH = 7.35). The perfusate was delivered using a roller pump (Cole Parmer Instrument Co, Niles, IL) at a constant flow rate at 8–14 mL/min warmed to 37±0.5°C. An air trap was used to avoid bubbles in the perfusion line.

### Wedge models of J wave syndrome

Transmembrane action potentials (AP) were recorded simultaneously from epicardial (Epi) and endocardial (Endo) regions of arterially-perfused canine LV and RV wedge preparations, together with a pseudo-ECG recorded across the bath. A detailed description of arterially-perfused ventricular wedge preparation has been published previously [9]. The preparations were stimulated from the endocardium, using bipolar silver electrodes insulated except at the tips at a 1000 ms cycle length. The Epi transmembrane microelectrodes, situated 0.5 cm apart, were used to obtain recordings at the very surface of the epicardium. At the time of arrhythmogenesis, the Epi electrodes were relocated to the triggering zone. The bipolar epicardial electrogram electrodes were placed in close proximity to the two Epi transmembrane microelectrodes.

We used a combination of I_to_-agonist NS5806 (3–10 μM) and calcium-channel (I_Ca_) blocker verapamil (0.5–2 μM) or the Class IA sodium-channel (I_Na_) blocker ajmaline (2–10 μM) to pharmacologically mimic the genetic defects and ion channel characteristics known to underlie ERS and BrS. The provocative agents were added to the coronary perfusate in increasing concentrations until the development of phase 2 reentry (P2R), electrogram (EG) abnormalities, closely coupled premature beats (PVCs) and PVT/VF. Following stable induction of ERS and BrS phenotypes, acacetin was added to the coronary perfusate.

### Patch clamp studies in HEK293 cells using polycistronic constructs

Patch clamp studies involving transfection of HEK293 cells with bicistronic constructs incorporating *SCN5A*-R878C and *KCND3*-WT or *SCN5A*-WT and *KCND3*-L450F associated with BrS and/or ERS were used to examine the reciprocal effects of these variants on I_to_ and to evaluate the effect of acacetin on inhibition of I_to_ generated under these pathologic conditions. These bicistonic plasmids were co-expressed with *KCNIP2*-WT+ RFP and *SCN1B*-WT+GFP.

The HEK cells were transfected with polycistronic constructs of R878C-*SCN5A+KCND3*+GFP and bicistronic constructs of *KCNIP2+RFP* and *SCN1B*+GFP to examine the effect of *R878C-SCN5A* variants to increase I_to_. We transfected the HEK cells with polycistronic constructs of L450F-*KCND3+SCN5A+GFP* and bicistronic constructs of *KCNIP2+RFP and SCN1B*+GFP to examine the effect of L450F-*KCND3* variants associated with BrS to reduce I_Na_. Patch-clamp macroscopic current recordings were carried out using whole-cell configuration at room temperature using Axopatch 200B amplifier as previously described [10,11]. To measure peak I_to_ amplitude and determine current–voltage relationships (I-V curves), currents were elicited using 600 ms-test potentials from -50 to +50 mV by increments of 10 mV from a holding potential of -90 mV. Following baseline measurements, I_to_ at +40 mV recordings was repeated during exposure to acacetin at concentrations ranging between 0.1 and 30 uM to calculate the IC_50_.

### Dissociation of canine ventricular myocytes

Single myocytes were obtained by enzymatic dissociation from coronary-perfused canine right ventricular wedge preparations. The RV wedge was coronary perfused with nominally Ca^+2^-free Tyrode’s solution for a period of 15 min. The wedge preparations were then subjected to enzymatic digestion in nominally Ca^+2^-free solution supplemented with 0.6 mg/ml collagenase (type II; Worthington) and 0.075 mg/ml protease (type XIV; Sigma) for 25–35 min. The wedge preparation was then perfused for 15 min with a taurine buffer solution containing (in mM): 108 NaCl, 10 HEPES, 11 D-glucose, 4 KCl, 1.2 MgSO_4_, 1.2 NaH_2_PO_4_, 50 Taurine and 25 nM of CaCl_2_. pH to 7.4. After perfusion, thin slices of tissue were cut from the epicardial surface to obtained single epicardial cells as previously described [12].

### Whole-cell patch clamp measurements of I_to_ and Action Potentials (APs) in dissociated canine right ventricular myocytes

I_to_ and APs were measured in isolated RV myocytes. APs were measured in the current-clamp mode in the whole-cell configuration. APs were initiated using intracellular current injection and recorded continuously by means of Clampex software (Axon Instruments/Molecular Devices) at a sampling rate of 1 pulse/2000 ms. I_to_ was measured using the Axopatch 200B-2 amplifier in V-Clamp mode.

I_to_ was measured in isolated RV epicardial cells at 37°C using whole-cell patch clamp techniques. I_to_ was elicited using a series of 350 ms voltage steps between -50 mV and +40 mV from a holding potential of -90 mV. A 15 ms prepulse to -20 mV was used to discharge the sodium current. Interpulse interval was varied from 2000 ms. The amplitude of I_to_ was be determined by subtracting peak current from steady-state current at the end of the pulse. The total charge carried by I_to_ was determined by calculating the area under the I_to_ current waveform. The extracellular solution contained (in mM): 140 NaCl, 4 KCl, 1.8 CaCl_2_, 1.2 MgCl_2_, 10 HEPES, and 10 glucose, pH = 7.4. The intracellular solution contained (in mM) 120 KCl, 1.5 MgCl_2_, 10 HEPES, 0.5 EGTA, 0.01 CaCl_2_, 5 MgATP, pH = 7.2. Following baseline measurements, recordings was repeated during exposure to acacetin (5 and 10 uM).

### Langendorff-perfused whole heart model of BrS and ERS

The ascending aorta was cannulated and secured with size zero silk suture. Retrograde aortic perfusion of ~1 liter of cold cardioplegic was performed at a constant pressure (60 mmHg; Langendorff mode) to fully rinse out the coronary vasculature. The atria were then removed.

The hearts were then connected to an organ bioreactor (Harvard Apparatus) and submerged in a 2.5-Liter chamber. Perfusion was performed in Langendorff-mode at a constant flow (400–500 ml/min) keeping the aortic pressure at 70±5 mmHg. The perfusate, a modified Krebs-Henseleit solution (K-H), was strongly bubbled with 95% O2/5% CO2. The composition of K-H was (in mM): 118 NaCl, 4 KCl, 2 CaCl_2_, 1.2 MgSO_4_, 24 NaHCO_3_, 1.2 NaH_2_PO_4_, 2 NaPyruvate and 10 D-Glucose. Unless otherwise specified, perfusate temperature and that of the heart chamber were kept at 37±0.5°C. Six liters of solution were recirculated for the duration of each experiment although, in some cases, no recirculation was performed.

The organ bioreactor system included a controller, a laptop, roller pumps, reservoirs, temperature sensors, pressure transducers, water heaters/circulator baths (Cole-Parmer), heating coils (Radnoti), in-line filter holders, a syringe pump (Harvard Apparatus) and a pulsar stimulator (FHC). The heart chamber contained multiple preset Ag/AgCl electrodes permitting simultaneous recordings of a 12-lead ECG with a 30-channel extracellular amplifier (Hugo Sachs Elektronik, Harvard Apparatus).

In addition to the ECG leads, up to 14 sub-epicardial (Epi) unipolar electrograms (U-EGs) were recorded with Ag/AgCl plunge electrode needles (Bruce Steadman and Alicja Booth; University of Utah, Salt Lake City) placed in the RVOT, RV inferior wall, LV antero-basal and LV apex. Each electrode was referenced to a bath ground (Ag/AgCl electrode). A unique feature of this model, although quite challenging, is the recording of intracellular transmembrane action potentials from the RVOT of vigorously beating hearts using floating glass microelectrodes. The hearts were either paced from the upper interventricular septum (just above the septal leaflet of the tricuspid valve) or allowed to beat spontaneously (junctional rhythm).

All amplified signals were digitized at a sample rate of 2,016 Hz, stored on magnetic media and analyzed using Spike 2 for Windows (Cambridge Electronic Design, Cambridge, UK).

#### Drugs

Acacetin, a derivative of the traditional Chinese medicine Xuelianhua, was synthesized in the laboratory of Gui-Rong Li or purchased from Sigma-Aldrich (St Louis, Mo). Acacetin was dissolved in dimethyl sulfoxide (100%) to create a 10 mM stock solution and stored at -20°C. Verapamil and ajmaline were also purchased from Sigma-Aldrich and were dissolved in distilled water and ethanol (100%), respectively. NS5806 was obtained from Gilead Sciences (Foster City, CA) or Sigma-Aldrich (St Louis, Mo), and was dissolved in dimethyl sulfoxide (100%).

#### Measurements and calculations

ECG-Parameters:

**QT**_**end**_: measured from the QRS-onset to the end of T wave (the point where the steepest tangent of the 2nd slope of the T wave meets the isoelectric line).

**QT**_**peak**_: measured from the QRS-onset to the apex of the T wave (the intersection between the steepest tangents of the two slopes of the T wave).

**T**_**peak**_**-T**_**end**_: measured from the apex to the end of T wave (see above).

**J wave area:** calculated using SigmaPlot software (Systat Software Inc.) and Excel. The onset of the J wave (J_o_) was set as defined by the recent consensus paper by Macfarlane et al [13, 14]. In case of J_o_< 0 (under the zero-line), the start of J wave was set to the point where its upslope meets the zero line. The end of the J wave (J_t_) was set to the point where its downslope meets the zero line (which was also the bottom limiting line for calculating the J wave area). For a better basis of comparison, in the wedge preparations (but not in the whole-heart models), J wave area was normalized to R wave amplitude (J wave area_r_), thus it was expressed as (mV_1_ x ms_1_)/mV_2_ units throughout the study, where mV_1_ x ms_1_ = area of the J wave and mV_2_ = R wave amplitude.

**QRS duration**: was measured from the onset of the QRS to the end of the S wave or to the J_o_.

Action Potential (AP) Parameters:

**AP notch area**: calculated using SigmaPlot software (Systat Software Inc). The start of the notch was defined as the peak of phase 0. The end of the notch was determined as the point where the AP reaches the top of the plateau (phase 2). The upper limiting line of the notch area was defined as the horizontal line set to the peak of phase 2 plateau. For a better basis of comparison, notch area was normalized to phase 2 amplitude (Notch area_r_), so thus it was expressed as (mV_1_ x ms_1_)/mV_2_ units throughout the study, where mV_1_ x ms_1_ = area of the AP notch and mV_2_ = phase 2 amplitude.

**Epicardial dispersion of repolarization (EDR)**: calculated as the longest interval between the epicardial AP durations at 90% of repolarization (APD_90_) in simultaneous recordings, corrected by the activation time (AT) as follows: EDR = (APD_90Epi1_ + AT_Epi1_)–(APD_90Epi2_ + AT_Epi2_).

**Transmural dispersion of repolarization (TDR)**: calculated as the longest interval between endocardial (Endo) and epicardial (Epi) APD_90_ values in simultaneous recordings, corrected by the AT as follows: TDR = (APD_90Endo_ + AT_Endo_)–(APD_90Epi_ + AT_Epi_).

#### Statistical analysis

Statistical analysis was performed using one-way repeated measures analysis of variance followed by all-pairwise comparisons using the Holm-Sidak method or linear regression, where appropriate. For the analysis of QT-parameters, paired t-test was used. Data are shown as mean ± SEM throughout the study. Statistical significance was considered at p<0.05.

## Results and discussion

**Fig 1:**
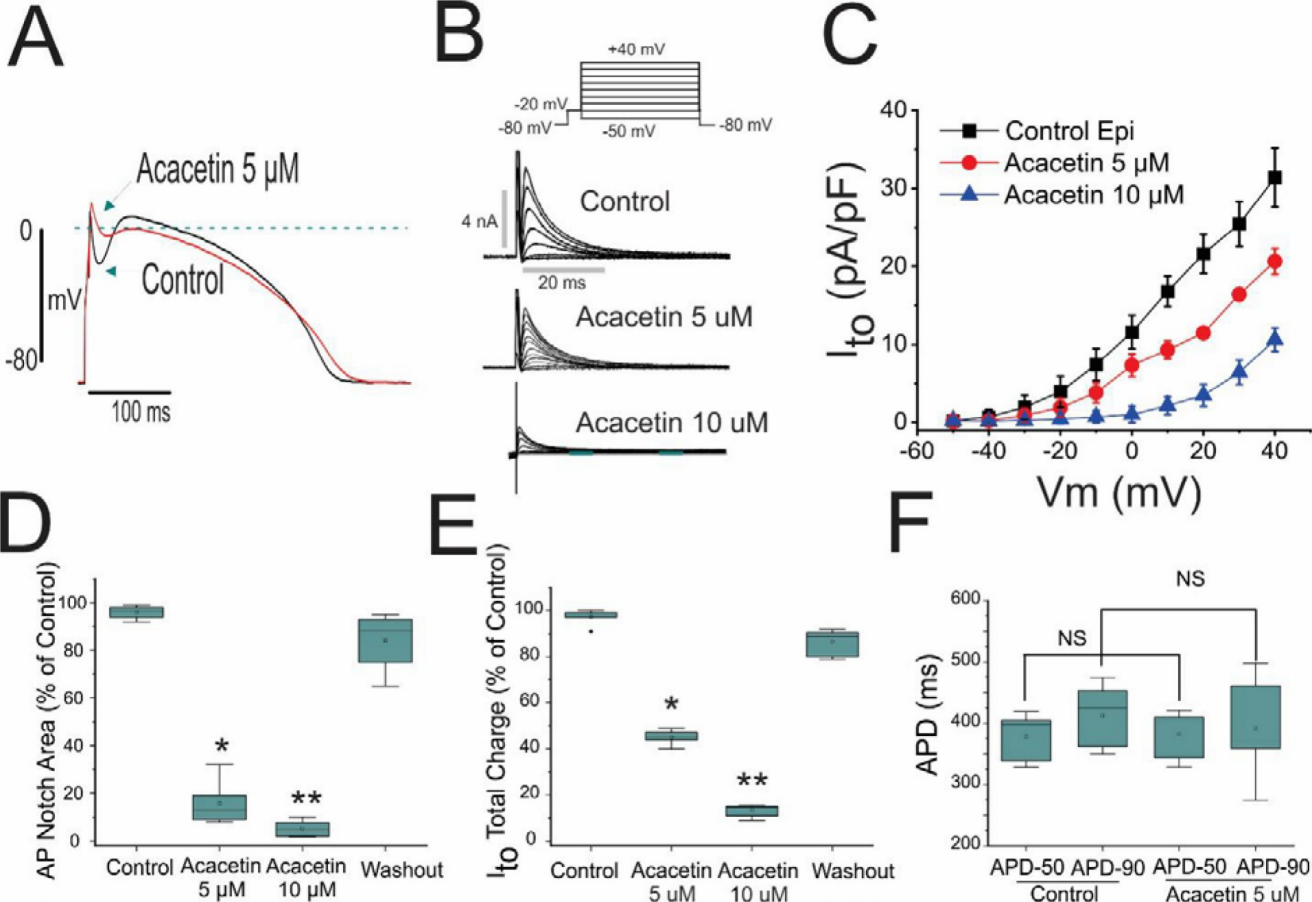
Effect of acacetin to inhibit the transient outward current (I_to_) and thus reduce the action potential notch. (A) Superimposed action potentials recorded from isolated canine ventricular myocyte before and after exposure to 5 μM acacetin. (B) Shown are traces of I_to_ recorded using the voltage protocol depicted in the inset before and after exposure to 5 and 10 μM acacetin. (C) I-V relationship for I_to_ density recorded from an isolated canine ventricular epicardial myocyte before and after 5 and 10 μM acacetin. (D) Area of action potential notch before and after 5 and 10 μM acacetin as well as after washout of the drug. (E) Total charge of I_to_ recorded at +20 mV before and after 5 and 10 μM acacetin as well as after washout of the drug. (F) Action potential duration at 50 and 90% repolarization recorded before and after 5 μM acacetin (n = 6); *p<0.05 and **p<0.01, respectively.

Fig 1 illustrates the effect of acacetin to inhibit I_to_ and to suppress the AP notch in epicardial myocytes enzymatically isolated from the canine RV, where I_to_ is known to be more prominent [15]. At a concentration of 5 and 10 μM, acacetin reduced I_to_ density by 56±2.1% and 80±3.2% with a proportional reduction in AP notch area. Action potential duration (APD) showed a tendency to abbreviate but was not significantly altered. Fig 1D–1F present composite data of the effect of acacetin on the AP notch, I_to_ total charge, and APD (APD_50 and 90_). Acacetin’s effect is predominantly on the early phases of the AP, consistent with a selective effect of the drug to inhibit I_to_.

**Fig 2:**
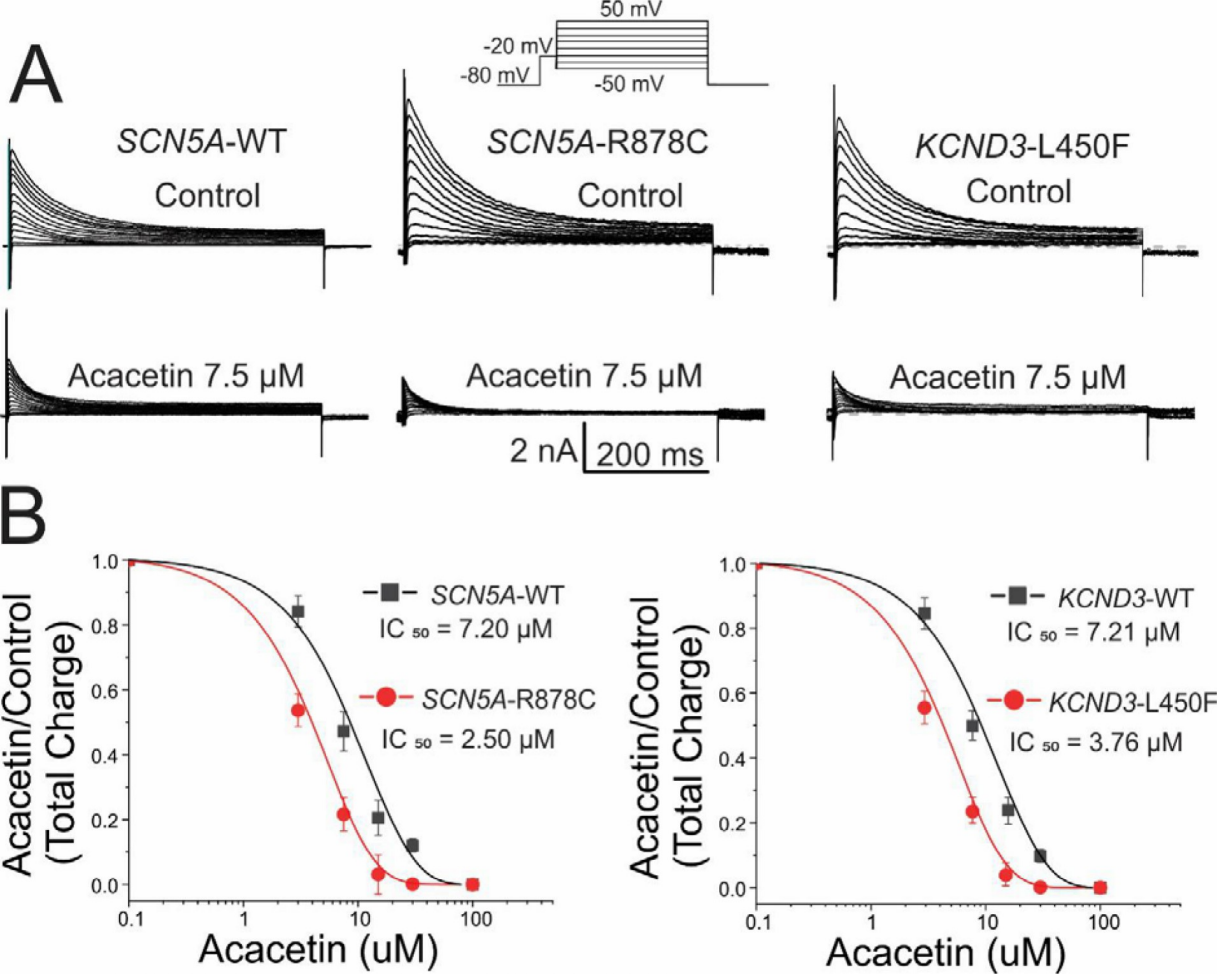
Pathology-upregulated I_to_ is more sensitive to inhibition by acacetin. Each panel shows I_to_ recorded from HEK293 cells transiently co-transfected with either WT- or R878C-SCN5A+KCND3, or SCN5A+WT or L450F KCND3, SCN1B and KChIP2 using polycistronic constructs. Voltage protocol is shown in the inset. (A) Representative traces of I_to_ and I_Na_. (B) Concentration-response relationships for I_to_ total charge (TC) normalized to the current recorded at +50 mV under control and acacetin conditions (room temperature). In the absence of JWS-causative variants, acacetin inhibited I_to_ with an IC_50_ of 7.2±1.0 μM-7.21±1.0 μM (n = 3) in HEK cells. In the presence of the JWS-causative variants, IC_50_ was reduced to 2.5±2 and 3.7±1.0 μM, respectively (Mean ±SEM; n = 3–5; p<0.05).

In recent studies (Clatot and Antzelevitch, unpublished observation), several BrS-associated *SCN5A* variants were shown to not only reduce I_Na_, but also to augment I_to_ and that *KCND3* variants associated with BrS and ERS can not only augment I_to_ but can also reduce I_Na_. In another series of experiments, we therefore evaluated the effect of acacetin to inhibit I_to_ augmented by these variants. Fig 2 illustrates that the effect of acacetin on this pathology-mediated I_to_ is more potent than on WT I_to_. In the absence of JWS-associated pathogenic variants, acacetin inhibited I_to_ with an IC_50_ of 7.2±1.0 μM (n = 3) when expressed in HEK cells, similar to the value obtained in native epicardial myocytes (6 μM). In the presence of the *SCN5A* and *KCND3* variants, IC_50_ was reduced to 2.5±2 and 3.7±1.0 μM, respectively (Mean ±SEM; n = 3–5; p<0.05).

**Fig 3:**
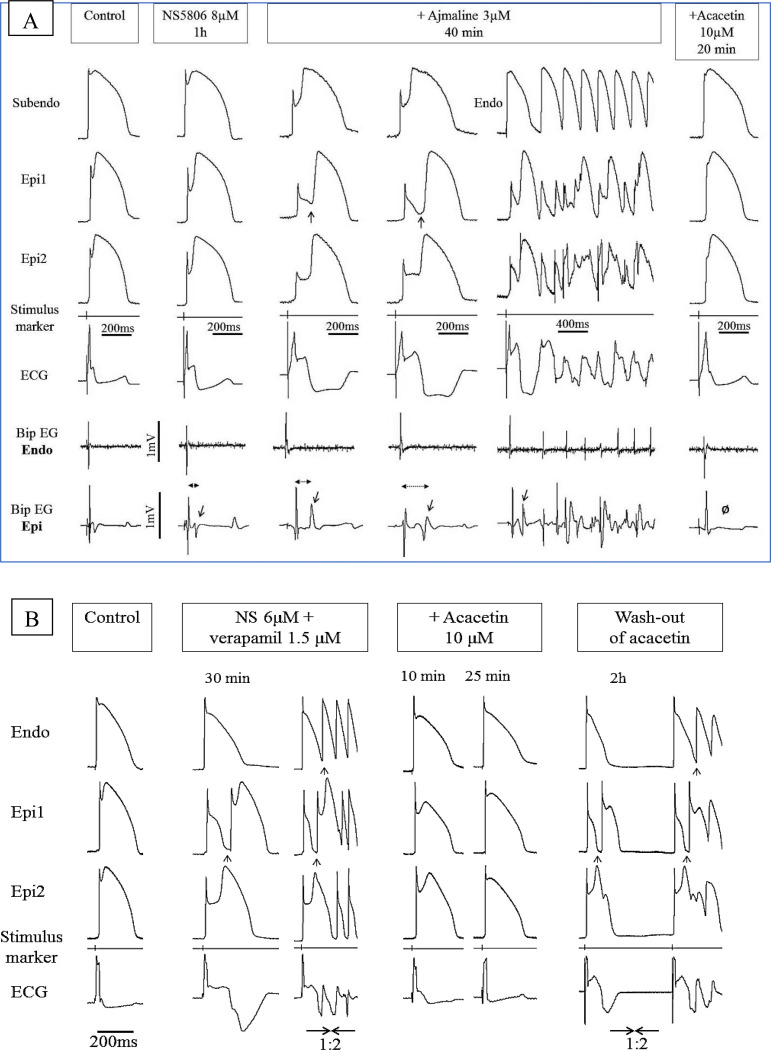
Effect of acacetin to suppress the electrocardiographic and arrhythmic manifestations in two different models of Brugada syndrome. Each panel shows simultaneously recorded transmembrane action potentials from one endocardial (Endo) or subendocardial (Subendo) and two epicardial (Epi1, Epi2) sites together with a pseudo-ECG and bipolar surface electrograms (Bip EG) in arterially-perfused RV wedge preparations. BCL = 1000 msec. (A) Brugada phenotype induced by a combination of NS5806 and ***ajmaline***. The provocative agents accentuated phase 1, leading to heterogeneous loss of the AP dome, thus giving rise to concealed P2R and a prominent type 1 ST segment elevation (col3). The delayed 2nd upstroke of the epicardial APs and concealed P2R give rise to late potentials in the bipolar electrograms recorded from epicardium but not endocardium (col2-4, Bip EG Epi and Bip EG Endo; red arrows). When P2R was able to propagate from its protected focus, it initiated VT/VF episodes (col5). Acacetin (10 μM) restored normal AP morphology secondary to diminution of the AP notch, thus abolishing all late potential activity (Ø), VT/VF, P2R and the Brugada- ECG-pattern (col6). (B) Brugada phenotype induced by the addition of NS5806 and ***verapamil*** to the coronary perfusate. The provocative agents accentuated phase 1, leading to heterogeneous loss of the AP dome in epicardium and the development of phase 2 reentry (col2-3, black arrows) and VT/VF (col3). Acacetin (10 μM) restored the epicardial AP dome, reduced the AP notch, and abolished all arrhythmic activity (col4-5). The effect of acacetin was reversed upon washout (col6).

In another series, we generated experimental models of BrS and ERS using canine ventricular RV and LV wedge preparations by pharmacologically mimicking the effects of BrS- and ERS-associated genetic defects, capable of causing an increase of I_to_ and/or reduction of either I_Na_ or I_Ca-L_. In the example illustrated in Fig 3, the addition to the coronary perfusate of the I_to_ agonist NS5806 (4–10 μM) dramatically augmented AP notch, much more so in epicardium (Epi) than in endocardium (Endo), resulting in accentuation of the J wave in the ECG, but without the development of arrhythmias (Fig 3A/col2). A delayed 2^nd^ upstroke of the AP was apparent in Epi resulting in a discrete delayed potential in the bipolar electrogram (EG) recorded from Epi (Fig 3A/col 2, red arrow). The addition of ajmaline (2–10 μM) or verapamil (0.5–2 μM) led to development of ERS (n = 6/6) and BrS (n = 6/8) phenotypes (Figs 3A/col3, 3B/col2 and 4/col2- and Table 1).

**Fig 4:**
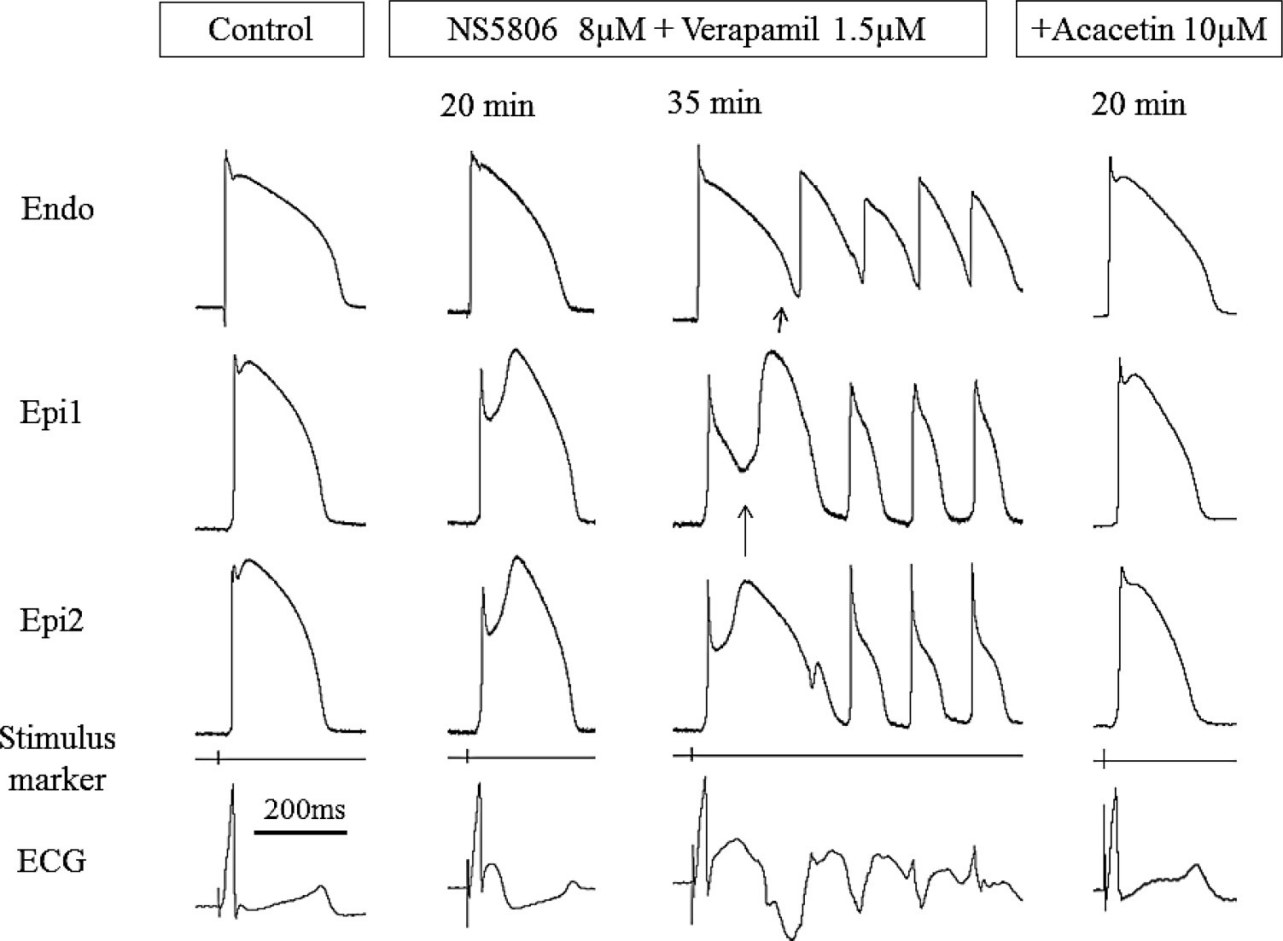
Effect of acacetin to suppress the electrocardiographic and arrhythmic manifestations in a model of ERS. Shown are simultaneous recordings of transmembrane action potentials from one endocardial (Endo) and two epicardial (Epi1, Epi2) sites together with a pseudo-ECG in arterially-perfused LV wedge preparations. BCL = 1000 msec. The combination of NS5806 and verapamil accentuated the epicardial AP notch leading to accentuated J waves, P2R and polymorphic VT (col2-3). Acacetin (10 μM) diminished the AP notch, restored AP dome, thus totally suppressing the J waves and VT (col4).

**Table 1:** Effect of acacetin on ECG and AP parameters in the two pharmacologic models of the J wave syndromes (series 2–3 experiments).

	*Control*	*Provocative Agents*	*+ Acacetin 10 μM*
*AP Notch area*_*r*_ *-RV*	6.32±1.46	73.77±9.23*	5.15±3.09*
*AP Notch area*_*r*_ *-LV*	2.56±0.52	60.73±7.39*	3.44±1.87*
*J wave area*_*r*_*-RV*	4.50±1.21	41.53±9.19*	3.33±1.37*
*J wave area*_*r*_*-LV*	1.48±0.59	37.76±6.09*	1.14±0.47*
*EDR-RV (ms)*	5.63±2.15	184.37± 23.93*	9.88±3.822*
*EDR-LV (ms)*	7.52±3.10	142.38± 17.08*	11.70±3.80*
*TDR-RV (ms)*	12.42±2.19	152.82±19.60*	13.45±3.93*
*TDR-LV (ms)*	10.63±5.52	116.73±12.25*	16.07±4.67*
*T*_*peak*_*-T*_*end*_*−RV (ms)*	26.30±2.49	130.57±15.51*	53.33±8.33*
*T*_*peak*_*-T*_*end*_*−LV (ms)*	39.75±2.53	97.87±14.53*	42.62±4.74*
*QT*_*peak*_*−RV (ms)*	229.23±6.71	259.99±18.21†	223.73±18.03†
*QT*_*peak*_*−LV (ms)*	227.07±3.05	192.52±39.43†	235.60±12.44†
*QT*_*end*_*−RV (ms)*	255.53±7.24	390.55±23.42*	277.07±16.92*
*QT*_*end*_*−LV (ms)*	266.82±2.71	289.80±27.48†	278.22±14.82†

*Control*: Recorded at baseline; *Provocative Agents*: After addition of NS5806 + verapamil or NS5806 + ajmaline to the perfusate; *+Acacetin 10 μM*: After addition of acacetin (10 μM) to the perfusate containing the provocative agents.

*p < 0.05

†p > 0.05 vs. previous experimental step; n = 6 for right (RV) and n = 6 for left ventricle (LV). No significant differences were found between the Control and the Acacetin groups.

The effect of acacetin to suppress the electrocardiographic and arrhythmic manifestations in a model of ERS is depicted in Fig 4 Shown are recordings of APs from one endocardial (Endo) and two epicardial (Epi1, Epi2) sites together with a transmural ECG in a LV wedge preparation. The combination of NS5806 and verapamil accentuated the Epi AP notch leading to accentuated J waves, P2R and polymorphic VT (col2-3). Acacetin (10 μM) diminished the AP notch, restored AP dome, thus totally suppressing the J waves and VT (col4).

**Fig 5:**
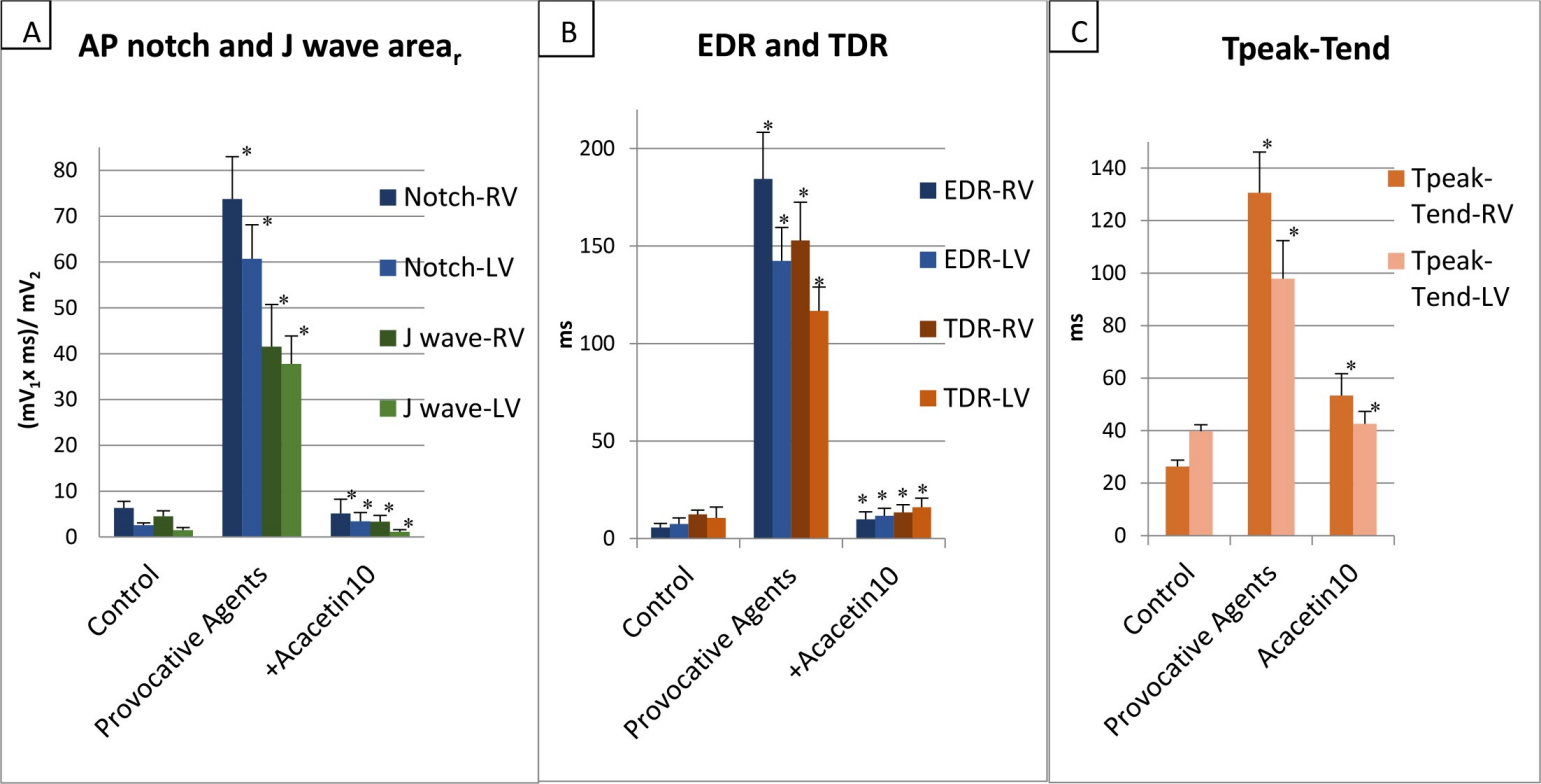
(A) Effect of acacetin on maximal action potential (AP) notch and J wave arear. (B) Epicardial and endocardial dispersion of repolarization (EDR and TDR). (C) Peak-to-end interval of the T wave (Tpeak-Tend) in the Brugada syndrome (RV) and early repolarization syndrome (LV) models. BCL = 1000 msec. Addition of the provocative agents created a prominent increase in all of these repolarization-dependent parameters, whereas acacetin reversed these changes and restored each value towards normal, irrespective of which pharmacologic model or which ventricle was used. All changes were significant compared to the previous experimental step. *p < 0.05 vs. previous experimental step; n = 6 for right (RV) and n = 6 for left ventricle (LV). No significant differences were found between the Control and the Acacetin groups.

These provocative agents dramatically increased the repolarization-mediated parameters in both RV and LV (Table 1 and Fig 5, n = 6 for RV and n = 6 for LV): Notch area increased by 1,067% and 2,269% in RV and LV, respectively (p<0.001 and p<0.001 vs. control) and J wave area increased by 823% and 2,460% in the RV and LV, respectively (p = 0.001 and p<0.001 vs control; Fig 5A and Table 1). There was a 3,173% and 1,794% increase in EDR (p<0.001 and p<0.001 vs. control, respectively) and 1,131% and 998% increase in the transmural dispersion of repolarization (TDR) compared to control in RV and LV, respectively (p<0.001 and p<0.001 vs. control; Fig 5B and Table 1). The provocative agents also increased Tpeak-Tend interval by 396% and 146% in RV and LV, respectively (p<0.001 and p = 0.003, vs. control; Fig 5C and Table 1).

The pronounced delay of the 2^nd^ AP upstroke, secondary to accentuation of the AP notch, and increased the epicardial dispersion of repolarization (EDR), due to heterogeneous loss of the AP dome, led to the development of phase 2 re-entry (P2R) giving rise to closely coupled PVCs in 12 out of 14 experiments, triggering polymorphic VT/VF in 11 of 14 preparations (Figs 3A/col3-5, 3B/col2-3 and 4/col2-3). Both ajmaline and verapamil augmented the fractionation of the bipolar EG and appearance of late potentials secondary to the repolarization abnormalities and development of concealed P2R (Fig 3A/col3-4, arrows). Interestingly, when ajmaline was applied to preparations displaying a very small AP notch and J wave at baseline, it reduced the manifestation of these parameters, due to the effect of these drugs to widen the QRS, thus engulfing the J wave. These observations are consistent with clinical reports describing attenuation of early repolarization ECG-pattern following ajmaline [16].

The addition of acacetin (10 μM) to the coronary perfusate dramatically suppressed both BrS and ERS phenotypes (Figs 3A/col6, 3B/col4-5 and 4/col4 and Table 1). AP notch and J wave area were reduced and the AP dome was restored in both LV (6/6) and RV (6/6), thus greatly reducing the dispersion of repolarization responsible for the development of P2R and VT/VF and eliminating fractionated EG activity and late potentials (Fig 5 and Table 1). All arrhythmic activity, including P2R, closely coupled extrasystoles and VT/VF were totally suppressed in all experiments (12/12) by 10 μM acacetin and in 7/8 experiments by 5 μM acacetin (Figs 3A/col6, 3B/col4-5 and 4/col4). The drug reversed the repolarization defects, but not the depolarization defects, induced by the provocative agents. QRS duration remained prolonged. In the RV wedge, the QRS duration before and after acacetin was 32.32±6.87 and 36.37±7.52 msec, respectively (Mean±SE [n = 6]; no significant differences). Similarly, in the LV wedge, the values were 37.08±5.01 and 41.07±4.93 msec, respectively (Mean±SE [n = 6]; no significant differences).

Acacetin decreased J wave area_r_ by 92% and 97% (p = 0.001 and p<0.001) and AP notch area_r_ by 93% and 94% in RV (n = 6) and LV (n = 6), respectively (p<0.001 and p<0.001; Fig 5A and Table 1). Acacetin 10 μM reduced EDR by 95% and 92% (p<0.001 and p<0.001), TDR was diminished by 91% and 86% (p<0.001 and p<0.001; Fig 5B and Table 1) whereas T_peak_-T_end_ decreased by 59% and 56% (p = 0.002 and p = 0.004; Fig 5C and Table 1) in RV and LV, respectively.

**Fig 6:**
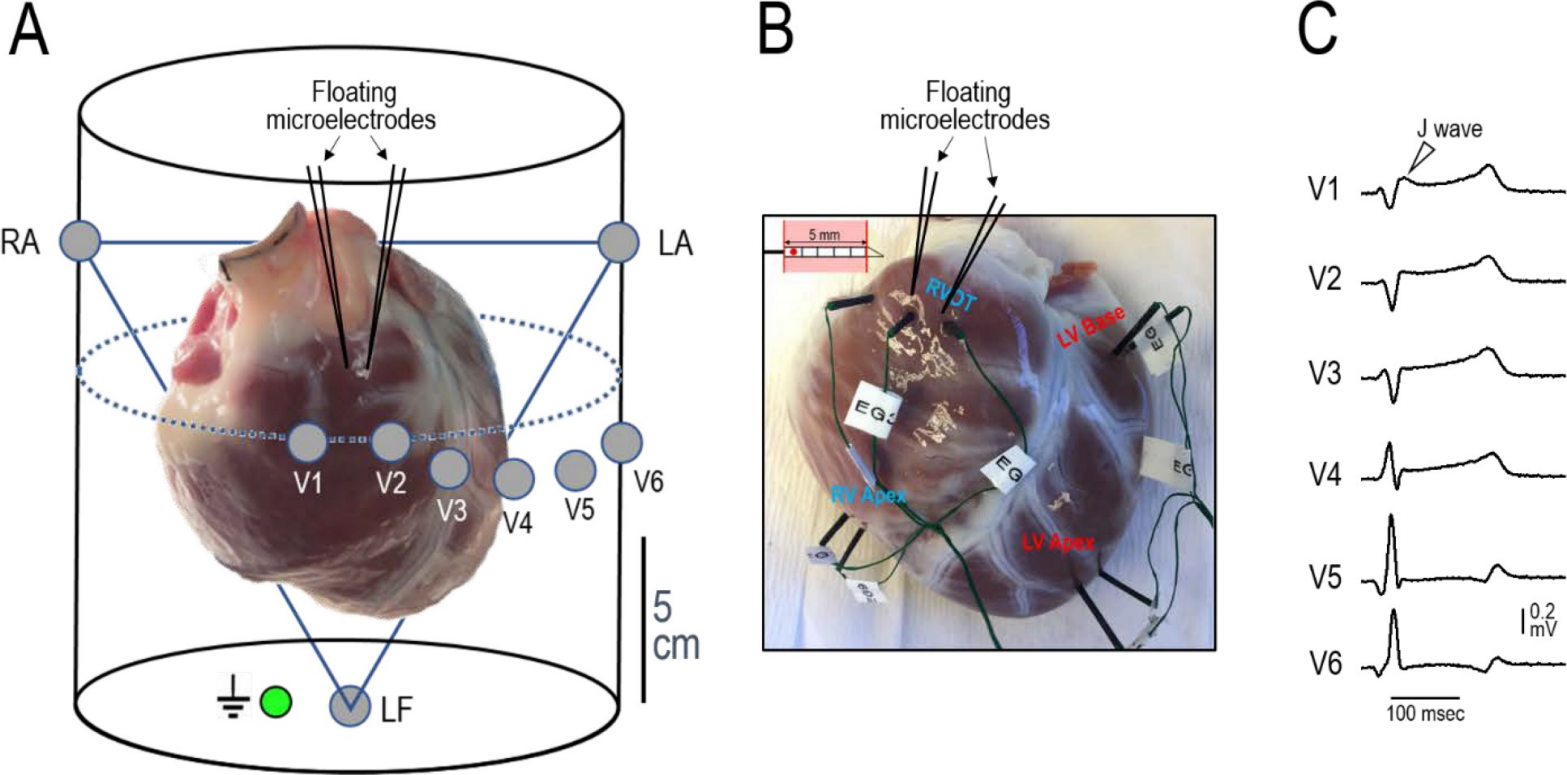
(A) Picture of a Langendorff-perfused canine heart (angled in its approximate anatomical position in the chest cavity) and schematic of the chamber used to record 12-lead ECGs. Whenever possible, one or two action potentials were recorded from the RVOT using floating microelectrodes. (B) Diagram of a plunge needle electrode used to record unipolar electrograms and their placement in the RVOT, Inferior RV and LV apex and base of a Langendorff-perfused heart. (C) Example of precordial ECG recordings obtained during pacing from the His bundle with a Medtronic pacing lead. Note the J wave displayed in lead V1.

**Fig 7:**
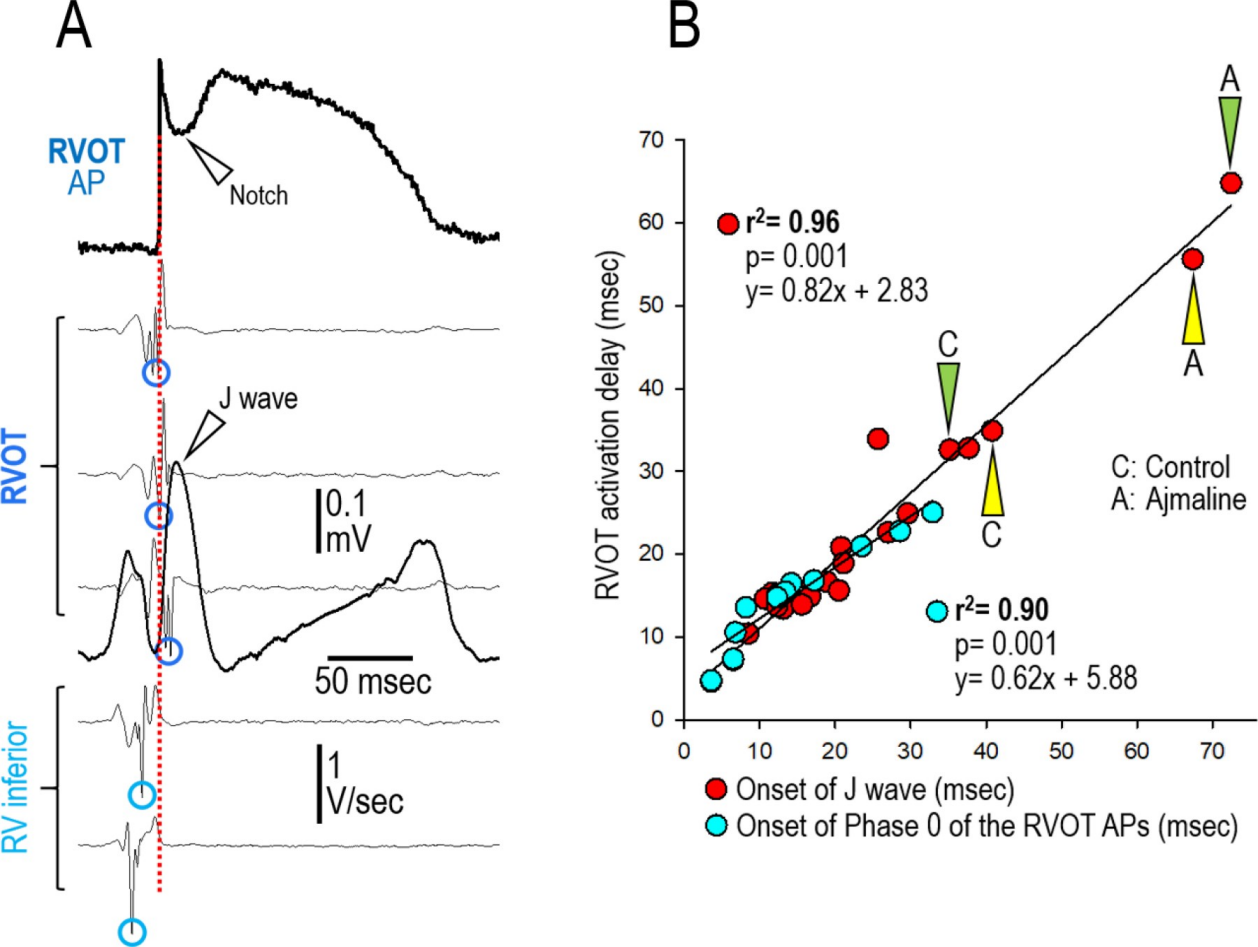
Correlation between RVOT activation delay (relative to the inferior RV), the onset of the J wave, and Phase 0 of epicardial APs recorded from the RVOT of a Langendorff-perfused whole heart model of the J wave syndrome. (A) From top to bottom: Transmembrane action potential recorded from the RVOT and lead V1 ECG superimposed on the first derivatives of simultaneously recorded Epicardial Unipolar electrograms (Epi Uni-EG) showing activation times (AT [V_min_]—depicted by circles) of the RVOT and the RV inferior wall. Recordings were obtained during junctional rhythm (CL = 860 msec). Note that Phase 0 of the RVOT action potential is in line with the onset of the J wave and the V_min_ of the RVOT Uni-EG; the vertical red dashed line highlights the correlation of these parameters. (B) Red circles: Correlation between the activation delay between the inferior RV and RVOT and the onset of the J wave (recorded in lead V1) under 22 different experimental conditions (11 Control, 6 NS5806 [5–7.5 μM], 3 Hypothermia [30–32°C] and 2 Ajmaline [10 μM]). Out of the 8 Langendorff-hearts included for this analysis, 5 were paced (BCLs = 500 [n = 1] and 800 msec [n = 4]) and 3 beat spontaneously (CLs: 790–2000 msec [Junctional Rhythm; JR]). Blue circles: Correlation between the RVOT activation delay and Phase 0 of the RVOT AP recorded under 11 experimental conditions (5 Control, 4 NS5806 [5–7.5 μM] and 2 Hypothermia [30–32°C]). Out of the 6 Langendorff-hearts included in this analysis, 4 were paced (BCLs = 500 [n = 2] and 800 msec [n = 2]) and 2 displayed a junctional rhythm (CLs: 790–2000 msec).

In another experimental series, we endeavored to create models of the JWS using Langendorff-perfused canine hearts to assess the validity of the results obtained in the wedge models of the JWS. Using this whole-heart model (Fig 6) we were able not only to validate the data obtained using the wed ge model but also to more critically define the mechanisms underlying the JWS. The cellular mechanisms underlying BrS, in particular, has long been a matter of controversy [17,18]. Two principle hypotheses have been advanced: (1) The **repolarization hypothesis** maintains that an outward shift in the balance of currents in the RV Epi leads to repolarization abnormalities, secondary to accentuation of the epicardial AP dome, resulting in the development of phase 2 reentry, which generates closely-coupled premature beats capable of precipitating VT/VF; (2) the **depolarization hypothesis** maintains that delayed conduction between RV and the RVOT plays a primary role in the development of the electrocardiographic and arrhythmic manifestations of the syndrome and that the J wave or ST segment elevation is due to a difference in activation time of RVOT vs. the remainder of RV. This hypothesis has never been tested in a whole-heart experimental model of BrS. According to the depolarization hypothesis, the latest activation of the RVOT should coincide with the **end** of the J wave, whereas in the repolarization hypothesis, it is expected to coincide with the **beginning** of the J wave. Fig 7A and 7B shows correlation of these parameters with the appearance of J waves induced under a variety of conditions including 1) augmentation of I_to_ using NS5806, 2) inhibition of I_Na_ using ajmaline and 3) hypothermia. In all cases, manifestation of the J wave coincided with the AP notch in RVOT Epi and its initiation (i.e., Phase 0 of its AP) coincides with activation of RVOT (as denoted by Vmin of the simultaneously recorded unipolar EGs), consistent with the repolarization hypothesis.

**Fig 8:**
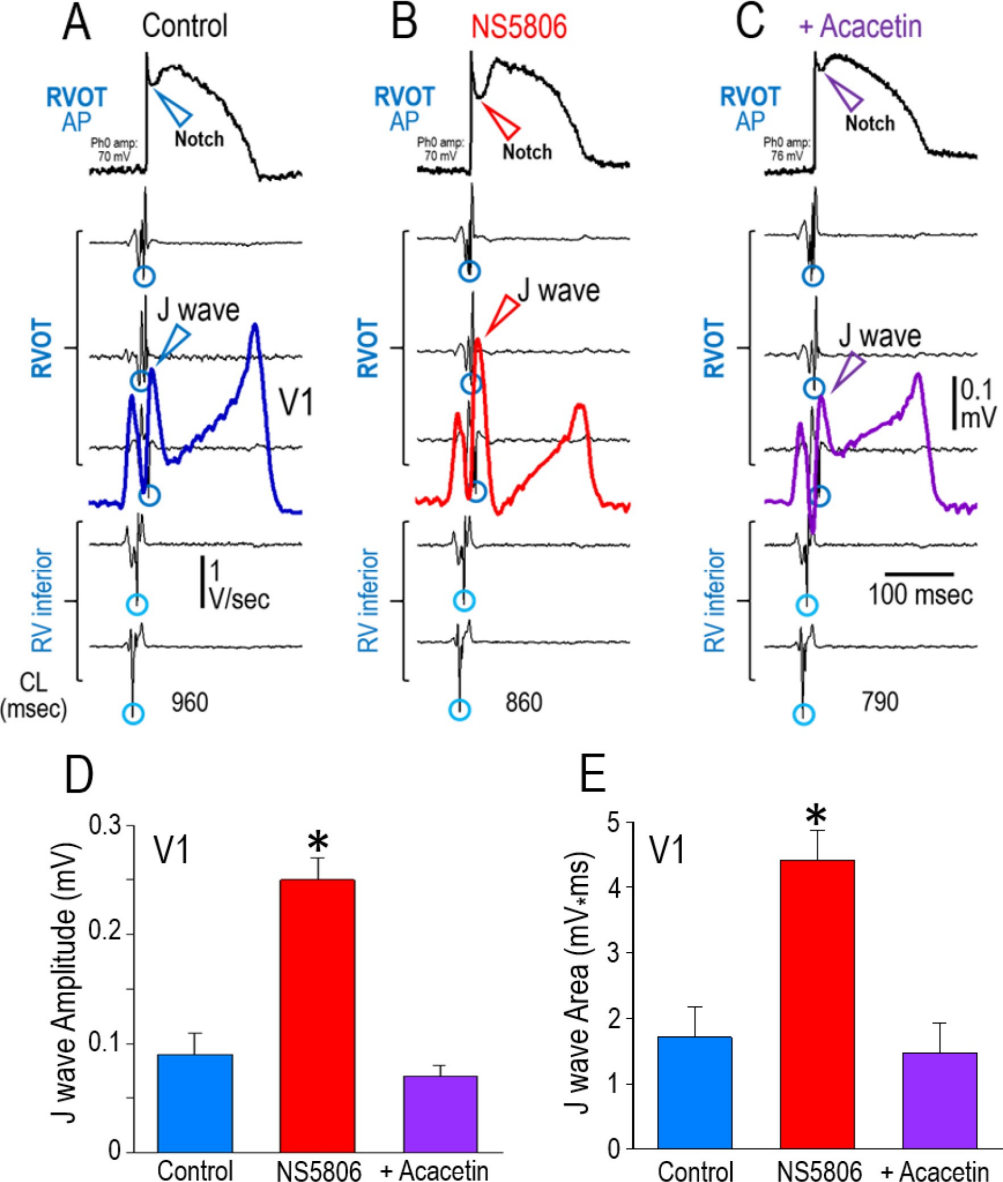
Effect of the I_to_ agonist NS5806 to amplify and acacetin to diminish the manifestation of the J wave in lead V1 and in the epicardial AP notch recorded from the RVOT of a Langendorff-perfused whole heart model of JWS. From top to bottom: Transmembrane APs recorded from the RVOT (unfiltered signals) and lead V1 superimposed on the first derivatives of the simultaneously recorded epicardial unipolar electrograms (U-EGs) showing activation times (V_min_−depicted by circles) of the RVOT and the RV inferior wall. All recordings were obtained during junctional rhythm (the atria were removed); the numbers at the bottom of each panel indicate the cycle length (CL) in msec. (A) Control. (B) 16 min after addition of NS5806 (7.5 μM).to the to the perfusate. (C) 8 min after addition of acacetin (5 μM; 8 min) significantly reduced the Epi AP notch and the corresponding J wave, thus suppressing arrhythmogenesis. (D) J wave **amplitude** recorded under Control conditions (Blue bar; n = 6), after exposure to NS5806 (Red bar; 5–7.5 μM [n = 6]) and after addition of acacetin (5 μM) to the perfusate (Purple bar; n = 6). Out of the 6 Langendorff-hearts included for this analysis, 3 were paced (BCLs = 800) and 3 exhibited junctional rhythm (CLs: 790–2000 msec). *p<0.001 between NS5806 and Control groups as well as that of NS5806 and Acacetin. No significant difference was found between the Control and the Acacetin groups. [Statistics: One Way Repeated Measures Analysis of Variance]. (E) J wave **area** recorded under the same experimental conditions shown in panel D. *p<0.001 between NS5806 and Control groups as well as that of NS5806 and Acacetin. No significant difference was found between the Control and the Acacetin groups. [Statistics: One Way Repeated Measures Analysis of Variance].

Fig 8 shows an example of the effect of the Ito agonist NS5806 to amplify, and acacetin to diminish, the manifestation of the J wave in lead V1 and in the epicardial AP notch recorded from the RVOT of a Langendorff-perfused whole-heart model of JWS. Panel A shows the Control recordings. NS5806 increased the amplitude of the J wave as well as the area under the J wave (Fig 8B). Addition of acacetin (5 μM) to the perfusate (Fig 8C) significantly reduced the J wave amplitude and the J wave area and prevented arrhythmogenesis in all 6 preparations exposed to acacetin. Panel D graphically illustrates the composite data for the J wave amplitude (n = 6; Mean±SE). The J wave amplitude increased from 0.090±0.02 mV (Control) to 0.25±0.02 mV (NS5806; p<0.001 vs. Control). Addition of acacetin to the perfusate reduced the amplitude of the J wave from 0.25±0.02 mV (NS5806) to 0.07±0.01 mV (Acacetin; p<0.001 vs. NS5806). Likewise, Panel E graphically illustrates the composite data for the area under the J wave (J wave area) from the same 6 experiments included in panel D. The J wave area increased from 1.71±0.47 mV* msec (Control) to 4.41±0.46 mV* msec (NS5806; p<0.001 [Mean±SE] vs. Control). Addition of acacetin to the perfusate reduced the J wave area from 4.41±0.46 mV* msec (NS5806) to 1.47±0.45 mV* msec (Acacetin; p<0.001 vs. NS5806).

**Fig 9:**
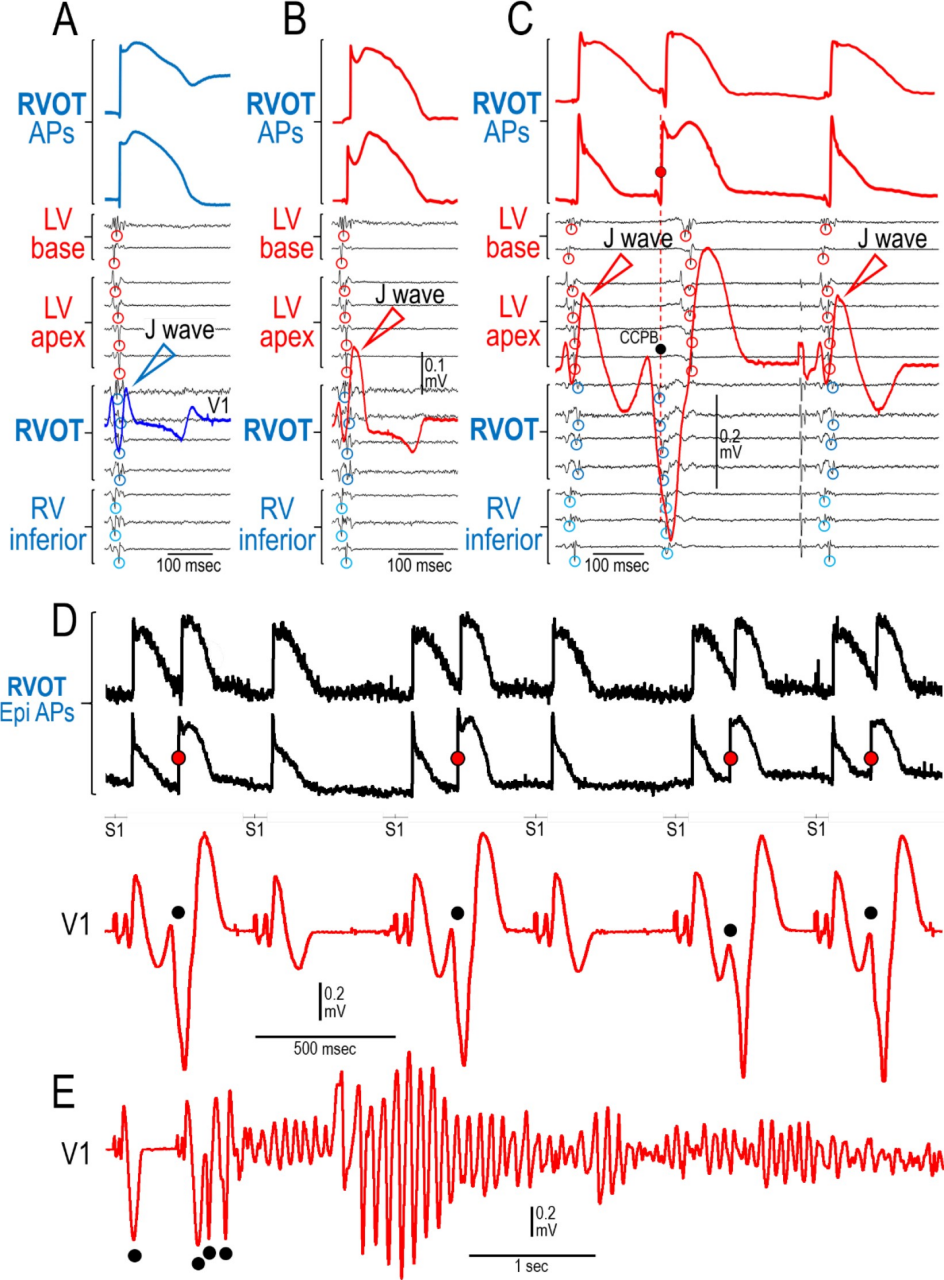
Closely coupled premature beats arising from the RVOT precipitate polymorphic VT in a Langendorff-perfused model of BrS. From top to bottom, shown are 2 action potentials simultaneously recorded from the epicardium of the RVOT and a lead V1 ECG superimposed on the first derivative of 13 Epi U-EGs recorded from the base and apex of the LV, the RVOT and the RV inferior wall (BCL = 500 msec). (A) Control. (B) Recorded 6 minutes after addition of the I_to_ activator NS5806 (7.5 μM) to the perfusate. NS5806 dramatically increased the amplitude and area of the J wave as well the magnitude and area of the notch of the action potential recorded form the epicardium of the RVOT. (C) Recorded after 23 minutes of exposure to NS5806. Shown are 2 consecutive paced beats (BCL = 500 msec). A closely coupled ventricular premature beat (CCPVB; denoted by a red [AP] and a black [V1] dot) follows the first paced response. The PVB arises from the RVOT (V_min_ of U-EG are circled). The first paced response shows loss of the Epi AP dome at one site but not the other creating an epicardial dispersion of repolarization (EDR). These CCPVBs arising from the RVOT and EDR are consistent with a phase 2 reentry mechanism. One minute later, multiple CCPBs precipitated VT/VF as shown in panels D and E. (D) Recorded after 23 minutes of exposure to NS5806 (7.5 μM); please note that the first 3 beats are the same 3 shown in panel C in which the AP signals were filtered (see Method section). Four CCPVBs are denoted by the red and black dots. (E) Recorded 80 sec after the traces depicted in panel D. Shown is the precipitation of polymorphic VT by 3 consecutive CCPVBs arising from the RVOT (lead V1). These electrophysiologic observations are consistent with a phase 2 reentry mechanism arising in the RVOT.

Fig 9 shows the ability of an increase in I_to_ mimicking a *KCND3*-mediated gain of function genetic variant to amplify the action potential notch in the Epi of the RVOT, giving rise to prominent J waves in the right precordial leads (shown is lead V1) and the development of epicardial dispersion of repolarization, thus creating the conditions for the development of phase 2 reentry in the RVOT. This results in the development of closely coupled extrasystoles arising from the RVOT, which precipitate polymorphic VT. Once again, it is clear that the appearance of the J wave is due to accentuation of early repolarization and not to delayed depolarization. As noted above, pre-treatment with 5 μM acacetin prevented the development of phase 2 reentry and closely coupled extrasystoles in 6 out of 6 preparations.

Our findings point to acacetin as a promising new agent for pharmacologic therapy of the JWS due to its unique effect to block I_to_ in the ventricular myocardium and thus prevent the repolarization abnormalities responsible for development of arrhythmogenesis in patients with the JWS. Acacetin may be especially advantageous in patients whose syndrome is accompanied by AF and/or ischemic events, in that it has been shown to exert an ameliorative effect in these settings [1,19,20]. Patients displaying early repolarization pattern (ERP) are generally more vulnerable to acute myocardial ischemia-related VT/VF [21,22]. In these individuals, in addition to its suppressive effects on arrhythmias associated with BrS and ERS, acacetin may protect against ischemia-reperfusion-mediated arrhythmogenesis [1,20].

Our study demonstrates the effect of acacetin, in concentrations as low as 5 μM, to suppress the electrocardiographic and arrhythmic manifestations of both BrS and ERS (7/8 cases). At a concentration of 10 μM, acacetin proved to be 100% effective in suppressing arrhythmogenesis in the JWS without exerting adverse electrophysiological effects. Acacetin also completely suppressed late potentials and fractionated electrogram activity. Our study adds to the long list of publications supporting the hypothesis that an inward shift in the balance of currents contributing to the early phases of the ventricular AP exerts ameliorative effects on the electrocardiographic and arrhythmic manifestations of the JWS.

Previous studies have shown that an increased T_peak_-T_end_ interval, an indicator of dispersion of repolarization [23], is associated with a higher risk for ventricular arrhythmias in BrS patients [24–26]. Our study provides further evidence in support of this hypothesis. The longest T_peak_-T_end_ intervals were observed at the time of initiation of arrhythmic activity and were characterized by the appearance of very negative T waves and P2R in both BrS and ERS models. It is noteworthy that such a dramatic increase in T_peak_-T_end_ is generally not observed in patients with ERS. Our findings suggest that this may be due to the fact that these electrocardiographic features are only observed for a brief period just before episodes of VT/VF. In support of this hypothesis are the findings of Nam et al. showing the development of negative T waves and prolonged Tpeak-Tend intervals in the right precordial ECG leads just before the occurrence of VT/VF in 5 patients diagnosed with ERS and electrical storms [27]. Letsas and co-workers also reported increased Tpeak-Tend in the right precordial leads of individuals with ERS [28]. In both of our BrS and ERS models, acacetin-mediated I_to_ block restored the ECG toward control values.

Acacetin (5 μM -10 μM) reportedly does not exert significant direct effects on other major currents in ventricular myocardium [1]. The slight tendency to abbreviate APD in the absence of provocative gents is expected due to the effect of acacetin to reduce or eliminate the AP notch.

The pathophysiology of BrS continues to be debated. The repolarization hypothesis maintains that an outward shift in the balance of currents during the early phases of the epicardial AP in the RVOT (where I_to_ is most prominent) underlies the accentuation of the J wave giving rise to the BrS ECG phenotype and abnormal repolarization leading to phase 2-reentry, which generates closely coupled premature beats capable of triggering VT/VF.

Several studies have suggested a primary role for conduction delay in the pathogenesis of JWS, especially BrS. [17,29,30] Our findings using the our novel whole-heart, Langendorff-perfused experimental model, indicate that a prominent activation delay in the RVOT is not required to explain the pathophysiological mechanism underlying the BrS ECG phenotype as proposed by the depolarization hypothesis. Even when activation delay between the RV inferior wall and the RVOT was induced by hypothermia or ajmaline, the onset of the J wave was always coincident with activation of the RVOT (inconsistent with the depolarization hypothesis) whereas inscription of the J wave was always directly associated with amplification of the AP notch recorded from the epicardium of the RVOT.

Our results are consistent with previous reports from our group and others showing that the J wave is due to expression of an I_to_-mediated AP notch in Epi but not Endo [31–33]. Past criticism was centered on the fact that the electrocardiographic and arrhythmic manifestations of the JWS were based on observations made in isolated wedge preparations and not in intact heart models. It was on this basis that the findings emanating from the wedge studies were rejected by many. These critics maintained that these observations would need to be validated in either an *in vivo* or whole heart models of the JWS in order to be considered as clinically relevant. Because *in vivo* models of the JWS are not available, we endeavored to generate an intact heart model.

Our whole-heart model of JWS shows that an outward shift in the balance of currents in the early phases of the Epi AP in the RVOT, produced by accentuation of I_to_ using NS5806 and/or inhibition of I_Na_ using ajmaline or with hypothermia, leads to an increase in RVOT Epi AP notch and corresponding amplification of the J wave, unrelated to delayed activation of the RVOT. The resulting BrS ECG phenotype, is associated with the appearance of closely-coupled extrasystoles originating from the RVOT, capable of triggering polymorphic VT/VF. These closely-coupled extrasystoles arise as a consequence of heterogeneous loss of the AP dome in the Epi of the RVOT, consistent with the development of phase 2-reentry.

The electrocardiographic and arrhythmic manifestations observed in our whole-heart model recapitulate the electrocardiographic and arrhythmic manifestations observed in patients with JWS. These findings validate those previously reported in coronary-perfused wedge models of BrS and ERS, providing support for the repolarization hypothesis. Our findings support the repolarization hypothesis proposed for the ECG manifestations of the BrS phenotype, but not the depolarization postulate. However, we acknowledge that the pathophysiologic mechanism(s) leading to development of life-threatening cardiac arrhythmias may involve a combination of those proposed by the two leading hypotheses (repolarization and depolarization).

Although conduction abnormality does not play a significant role in our models [34], we would like to emphasize that our study is not intended to prove the exclusivity of the repolarization hypothesis. Our findings do not deny the possible contribution of slow or delayed conduction to the development of arrhythmogenesis in BrS (or ERS). Rather, these investigations provide support for the hypothesis that, in experimental models that mimic the phenotypic manifestations of genetic factors and ion channel heterogeneities responsible for JWS, repolarization abnormalities alone are capable of recapitulating all features of the JWS observed clinically.

Acacetin exerted a potent ameliorative effect regardless of whether the JWS phenotype was generated using a gain of function of outward currents (**Figs 7 and 8**) or a loss of function of inward currents (**Figs 3A and 3B and 4**).

### Study limitations

Acacetin has diverse biological activity in that it has been shown to be hepatoprotective, neuroprotective, immunomodulatory, anticancer [35–40], anti-inflammatory [35,41–43], antipyretic-antinociceptive [35,44], aromatase-inhibitory [45], antioxidant and antibiotic [46] in its actions. Despite this broad testing and application, acacetin is reported to have very low or no acute toxicity in experimental studies [1].

While the pharmacological models that we employ may not precisely mimic the effect of the diverse oligogenic genetic variants underlying the clinical syndromes, “more realistic” transgenic large mammal models do not exist. Our observations using the wedge models over 20 years ago suggested for the first time that an early repolarization pattern in the ECG, previously thought to be totally benign, can reveal the presence of a substrate for the development of malignant arrhythmias [2], and identified novel approaches to therapy of both BrS and ERS [47].

It can also be argued that arterially-perfused wedge preparations do not represent the complete anatomical structure of the heart and thus may not fully recapitulate the disease phenotype. The arterially perfused canine ventricular wedge model is capable of recapitulating all features of the BrS and ERS (e.g. the response to pharmacologic agents, to ablation, to changes in heart rate, as well as electrocardiographic and arrhythmic manifestations). These models permitted us and other groups the ability to elucidate the cellular mechanisms and thus to recommend novel therapeutic approaches, including the use of quinidine and isoproterenol for the treatment of the JWS [4], which are widely used in the clinic today to deal with JWS-associated electrical storms or as an adjunct to ICD therapy. In subsequent studies, these same techniques were used to demonstrate the effectiveness of cilostazol and milrinone [48]. Cilostazol, quinidine, and isoproterenol are all part of the recommended therapy included in the recent expert consensus conference report [3]. These models have also identified ECG markers such as Tpeak-Tend and QT/RR relationships that have proved useful in risk stratification of patients with inherited cardiac arrhythmia syndromes [49–52]. Finally, the present study provides important validation of the results obtained in the wedge models by demonstrating these same mechanisms in the whole-heart model of the JWS.

## References

[1] Li GR, Wang HB, Qin GW, Jin MW, Tang Q, Sun HY, et al Acacetin, a natural flavone, selectively inhibits human atrial repolarization potassium currents and prevents atrial fibrillation in dogs. Circulation. 2008;117(19):2449–57. 10.1161/CIRCULATIONAHA.108.769554 18458165

[2] Antzelevitch C, Yan GX. J wave syndromes. Heart Rhythm. 2010;7(4):549–58. 10.1016/j.hrthm.2009.12.006 20153265 PMC2843811

[3] Antzelevitch C, Yan GX, Ackerman MJ, Borggrefe M, Corrado D, Guo J, et al J-Wave syndromes expert consensus conference report: Emerging concepts and gaps in knowledge. Heart Rhythm. 2016 Epub 2016/07/18. 10.1016/j.hrthm.2016.05.024 . 27423412 PMC5035208

[4] Yan GX, Antzelevitch C. Cellular basis for the Brugada syndrome and other mechanisms of arrhythmogenesis associated with ST-segment elevation. Circulation. 1999;100(15):1660–6. 10.1161/01.cir.100.15.1660 10517739

[5] Koncz I, Gurabi Z, Patocskai B, Panama BK, Szel T, Hu D, et al Mechanisms underlying the development of the electrocardiographic and arrhythmic manifestations of early repolarization syndrome. Journal of molecular and cellular cardiology. 2014;68C:20–8. 10.1016/j.yjmcc.2013.12.012 24378566 PMC3943882

[6] Giustetto C, Cerrato N, Gribaudo E, Scrocco C, Castagno D, Richiardi E, et al Atrial fibrillation in a large population with Brugada electrocardiographic pattern: prevalence, management, and correlation with prognosis. Heart Rhythm. 2014;11(2):259–65. 10.1016/j.hrthm.2013.10.043 . 24513919

[7] Bordachar P, Reuter S, Garrigue S, Cai X, Hocini M, Jais P, et al Incidence, clinical implications and prognosis of atrial arrhythmias in Brugada syndrome. EurHeart J. 2004;25(10):879–84. 10.1016/j.ehj.2004.01.004 15140537

[8] Antzelevitch C, Patocskai B. Brugada Syndrome: Clinical, Genetic, Molecular, Cellular, and Ionic Aspects. Curr Probl Cardiol. 2016;41(1):7–57. Epub 2015/12/17. 10.1016/j.cpcardiol.2015.06.002 26671757 PMC4737702

[9] Di Diego JM, Sicouri S, Myles RC, Burton FL, Smith GL, Antzelevitch C. Optical and electrical recordings from isolated coronary-perfused ventricular wedge preparations. Journal of molecular and cellular cardiology. 2013;54(1):53–64. 10.1016/j.yjmcc.2012.10.017 23142540 PMC3535682

[10] Hu D, Barajas-Martinez H, Kahlig K, Rajamani S, Belardinelli L, Pfeiffer R, et al Genetic variants in SCN10A associated with Brugada sydrome, right bundle branch block and atrioventricular block. Heart Rhythm. 2012;9:S395.

[11] Barajas-Martinez H, Smith M, Hu D, Goodrow RJ, Puleo C, Hasdemir C, et al Susceptibility to Ventricular Arrhythmias Resulting from Mutations in FKBP1B, PXDNL, and SCN9A Evaluated in hiPSC Cardiomyocytes. Stem cells international. 2020;2020:8842398 Epub 2020/09/22. 10.1155/2020/8842398 32952569 PMC7481990

[12] Barajas-Martinez H, Haufe V, Chamberland C, Blais Roy MJ, Fecteau MH, Cordeiro JM, et al Larger dispersion of INa in female dog ventricle as a mechanism for gender-specific incidence of cardiac arrhythmias. CardiovascRes. 2009;81(1):82–9. 10.1093/cvr/cvn255 18805783

[13] Macfarlane P.; Antzelevitch C.; Haissaguerre M.; Huikuri HPMRR, Sacher F.; Tikkanen J.; et al Consensus Paper- Early Repolarization Pattern. J Amer Coll Cardiol. 2015;66(4):470–7. 10.1016/j.jacc.2015.05.033 26205599

[14] Macfarlane PW, Antzelevitch C, Haissaguerre M, Huikuri HV, Potse M, Rosso R, et al The early repolarization pattern: A consensus paper. J Am Coll Cardiol. 2015;66(4):470–7. Epub 2015/07/25. 10.1016/j.jacc.2015.05.033 . 26205599

[15] Di Diego JM, Sun ZQ, Antzelevitch C. Ito and action potential notch are smaller in left vs. right canine ventricular epicardium. AmJPhysiol. 1996;271:H548–H61. 10.1152/ajpheart.1996.271.2.H548 8770096

[16] Roten L, Derval N, Sacher F, Pascale P, Wilton SB, Scherr D, et al Ajmaline attenuates electrocardiogram characteristics of inferolateral early repolarization. Heart Rhythm. 2012;9(2):232–9. 10.1016/j.hrthm.2011.09.013 21914496

[17] Wilde AA, Postema PG, Di Diego JM, Viskin S, Morita H, Fish JM, et al The pathophysiological mechanism underlying Brugada syndrome: depolarization versus repolarization. Journal of molecular and cellular cardiology. 2010;49(4):543–53. 10.1016/j.yjmcc.2010.07.012 20659475 PMC2932806

[18] Morita H, Miura D, Nishii N, Nagase S, Nakamura K, Kusano KF, et al Differential effects of genotypeon the initiation of ventricular arrhythmias in patients with Brugada syndrome. Heart Rhythm. 2009;6:S349 10.1016/j.hrthm.2009.01.031 19324308

[19] Wu HJ, Sun HY, Wu W, Zhang YH, Qin GW, Li GR. Properties and molecular determinants of the natural flavone acacetin for blocking hKv4.3 channels. PloS one. 2013;8(3):e57864 10.1371/journal.pone.0057864 23526953 PMC3603988

[20] Yang WJ, Liu C, Gu ZY, Zhang XY, Cheng B, Mao Y, et al Protective effects of acacetin isolated from Ziziphora clinopodioides Lam. (Xintahua) on neonatal rat cardiomyocytes. Chin Med. 2014;9(1):28 Epub 2015/01/06. 10.1186/s13020-014-0028-3 25558275 PMC4272544

[21] Naruse Y, Tada H, Harimura Y, Ishibashi M, Noguchi Y, Sato A, et al Early repolarization increases the occurrence of sustained ventricular tachyarrhythmias and sudden death in the chronic phase of an acute myocardial infarction. Circ ArrhythmElectrophysiol. 2014;7(4):626–32. 10.1161/CIRCEP.113.000939 24863485

[22] Tikkanen JT, Wichmann V, Junttila MJ, Rainio M, Hookana E, Lappi OP, et al Association of early repolarization and sudden cardiac death during an acute coronary event. Circ Arrhythm Electrophysiol. 2012;5(4):714–8. 10.1161/CIRCEP.112.970863 22730409

[23] Antzelevitch C. T peak-Tend interval as an index of transmural dispersion of repolarization. EurJClinInvest. 2001;31(7):555–7. 10.1046/j.1365-2362.2001.00849.x 11454006

[24] Castro Hevia J, Antzelevitch C, Tornes Barzaga F, Dorantes Sanchez M, Dorticos Balea F, Zayas Molina R, et al Tpeak-Tend and Tpeak-Tend dispersion as risk factors for ventricular tachycardia/ventricular fibrillation in patients with the Brugada syndrome. J Am Coll Cardiol. 2006;47(9):1828–34. Epub 2006/05/10. 10.1016/j.jacc.2005.12.049 16682308 PMC1474075

[25] Lambiase PD. Tpeak-Tend interval and Tpeak-Tend/QT ratio as markers of ventricular tachycardia inducibility in subjects with Brugada ECG phenotype. Europace. 2010;12(2):158–9. Epub 2010/01/05. 10.1093/europace/eup424 . 20045864

[26] Maury P, Sacher F, Gourraud JB, Pasquie JL, Raczka F, Bongard V, et al Increased Tpeak-Tend interval is highly and independently related to arrhythmic events in Brugada syndrome. Heart Rhythm. 2015;12(12):2469–76. Epub 2015/07/26. 10.1016/j.hrthm.2015.07.029 . 26209263

[27] Nam GB, Ko KH, Kim J, Park KM, Rhee KS, Choi KJ, et al Mode of onset of ventricular fibrillation in patients with early repolarization pattern vs. Brugada syndrome. Eur Heart J. 2010;31(3):330–9. 10.1093/eurheartj/ehp423 19880418 PMC2814221

[28] Letsas KP, Charalampous C, Korantzopoulos P, Tsikrikas S, Bramos D, Kollias G, et al Novel indexes of heterogeneity of ventricular repolarization in subjects with early repolarization pattern. Europace. 2012;14(6):877–8. 10.1093/europace/eur390 22186777

[29] Hoogendijk MG, Coronel R. The J-wave conundrum: early repolarization and Brugada syndrome. Heart Rhythm. 2013;10(4):540–1. Epub 2013/01/15. 10.1016/j.hrthm.2013.01.005 . 23313799

[30] Nademanee K, Raju H, de Noronha SV, Papadakis M, Robinson L, Rothery S, et al Fibrosis, Connexin-43, and Conduction Abnormalities in the Brugada Syndrome. J Am Coll Cardiol. 2015;66(18):1976–86. Epub 2015/10/31. 10.1016/j.jacc.2015.08.862 26516000 PMC4631798

[31] Litovsky SH, Antzelevitch C. Transient outward current prominent in canine ventricular epicardium but not endocardium. CircRes. 1988;62(1):116–26. 10.1161/01.res.62.1.116 2826039

[32] Litovsky SH, Antzelevitch C. Differences in the electrophysiology of ventricular epicardium and endocardium as the basis for the Osborne wave. Circulation. 1989;80:II–129.

[33] Yan GX, Antzelevitch C. Cellular basis for the electrocardiographic J wave. Circulation. 1996;93(2):372–9. 10.1161/01.cir.93.2.372 8548912

[34] Patocskai B, Yoon N, Antzelevitch C. Mechanisms underlying epicardial radiofrequency ablation to suppress arrhythmogenesis in experimental models of Brugada syndrome. JACC: Clinical Electrophysiology. 2017;3(4):353 10.1016/j.jacep.2016.10.011 28948234 PMC5609479

[35] Liao YH, Houghton PJ, Hoult JR. Novel and known constituents from Buddleja species and their activity against leukocyte eicosanoid generation. J Nat Prod. 1999;62(9):1241–5. Epub 1999/10/09. 10.1021/np990092+ . 10514305

[36] Pan MH, Lai CS, Wang YJ, Ho CT. Acacetin suppressed LPS-induced up-expression of iNOS and COX-2 in murine macrophages and TPA-induced tumor promotion in mice. Biochem Pharmacol. 2006;72(10):1293–303. Epub 2006/09/05. 10.1016/j.bcp.2006.07.039 . 16949556

[37] Shim HY, Park JH, Paik HD, Nah SY, Kim DS, Han YS. Acacetin-induced apoptosis of human breast cancer MCF-7 cells involves caspase cascade, mitochondria-mediated death signaling and SAPK/JNK1/2-c-Jun activation. Mol Cells. 2007;24(1):95–104. Epub 2007/09/12. . 17846503

[38] Chien ST, Lin SS, Wang CK, Lee YB, Chen KS, Fong Y, et al Acacetin inhibits the invasion and migration of human non-small cell lung cancer A549 cells by suppressing the p38alpha MAPK signaling pathway. Mol Cell Biochem. 2011;350(1–2):135–48. Epub 2011/01/07. 10.1007/s11010-010-0692-2 . 21210297

[39] Watanabe K, Kanno S, Tomizawa A, Yomogida S, Ishikawa M. Acacetin induces apoptosis in human T cell leukemia Jurkat cells via activation of a caspase cascade. Oncol Rep. 2012;27(1):204–9. Epub 2011/10/14. 10.3892/or.2011.1498 . 21993665

[40] Kim HR, Park CG, Jung JY. Acacetin (5,7-dihydroxy-4'-methoxyflavone) exhibits in vitro and in vivo anticancer activity through the suppression of NF-kappaB/Akt signaling in prostate cancer cells. International journal of molecular medicine. 2014;33(2):317–24. Epub 2013/11/29. 10.3892/ijmm.2013.1571 . 24285354

[41] Aguilar-Shea AL, Gallardo-Mayo C. A case of Brugada syndrome. QJM: monthly journal of the Association of Physicians. 2015;108(3):235–7. 10.1093/qjmed/hcs127 . 22753674

[42] Huang WC, Liou CJ. Dietary acacetin reduces airway hyperresponsiveness and eosinophil infiltration by modulating eotaxin-1 and th2 cytokines in a mouse model of asthma. Evid Based Complement Alternat Med. 2012;2012:910520 Epub 2012/10/11. 10.1155/2012/910520 23049614 PMC3462452

[43] Srisook K, Srisook E, Nachaiyo W, Chan-In M, Thongbai J, Wongyoo K, et al Bioassay-guided isolation and mechanistic action of anti-inflammatory agents from Clerodendrum inerme leaves. J Ethnopharmacol. 2015;165:94–102. Epub 2015/03/01. 10.1016/j.jep.2015.02.043 . 25725433

[44] Rauf A, Khan R, Khan H, Ullah B, Pervez S. Antipyretic and antinociceptive potential of extract/fractions of Potentilla evestita and its isolated compound, acacetin. BMC Complement Altern Med. 2014;14:448 Epub 2014/11/20. 10.1186/1472-6882-14-448 25407486 PMC4247777

[45] Zhao J, Dasmahapatra AK, Khan SI, Khan IA. Anti-aromatase activity of the constituents from damiana (Turnera diffusa). J Ethnopharmacol. 2008;120(3):387–93. Epub 2008/10/25. 10.1016/j.jep.2008.09.016 . 18948180

[46] Komape NP, Aderogba M, Bagla VP, Masoko P, Eloff JN. Anti-bacterial and anti-oxidant activities of leaf extracts of Combretum vendae (Combretecacea) and the isolation of an anti-bacterial compound. Afr J Tradit Complement Altern Med. 2014;11(5):73–7. Epub 2014/11/15. 10.4314/ajtcam.v11i5.12 25395708 PMC4202521

[47] Yan GX, Antzelevitch C. Cellular basis for the Brugada syndrome and other mechanisms of arrhythmogenesis associated with ST segment elevation. Circulation. 1999;100(15):1660–6. 10.1161/01.cir.100.15.1660 10517739

[48] Gurabi Z, Koncz I, Patocskai B, Nesterenko VV, Antzelevitch C. Cellular mechanism underlying hypothermia-induced ventricular tachycardia/ventricular fibrillation in the setting of early repolarization and the protective effect of quinidine, cilostazol, and milrinone. Circ Arrhythm Electrophysiol. 2014;7(1):134–42. Epub 2014/01/17. 10.1161/CIRCEP.113.000919 24429494 PMC3951442

[49] Antzelevitch C. The role of spatial dispersion of repolarization in inherited and acquired sudden cardiac death syndromes. Am J Physiol Heart CircPhysiol. 2007;293(4):H2024–H38. 10.1152/ajpheart.00355.2007 17586620 PMC2085107

[50] Extramiana F, Antzelevitch C. Amplified transmural dispersion of repolarization as the basis for arrhythmogenesis in a canine ventricular-wedge model of short QT syndrome. Circulation. 2004;110(24):3661–6. 10.1161/01.CIR.0000143078.48699.0C 15569843

[51] Patel C, Antzelevitch C. Cellular basis for arrhythmogenesis in an experimental model of the SQT1 form of the short QT syndrome. Heart Rhythm. 2008;5(4):585–90. 10.1016/j.hrthm.2008.01.022 18362027 PMC2361425

[52] Antzelevitch C, Guerchicoff A, Pollevick GD. The role of spatial dispersion of repolarization in sudden cardiac death. 2006.

